# Development and Preliminary Psychometric Evaluation of the ACRIS-CS: A Theory-Based Measure of Prospective Acceptability of Cognitive Rehabilitation in Cancer Survivors

**DOI:** 10.3390/jcm15051858

**Published:** 2026-02-28

**Authors:** Ana F. Oliveira, Ana Bártolo, Liliana Loureiro, Helena Sousa, Ana Torres, Isabel M. Santos

**Affiliations:** 1 RISE-Health: Health Research Network, Department of Education and Psychology, University of Aveiro, Campus Universitário de Santiago, 3810-193 Aveiro, Portugal; helena.sousa@ua.pt; 2 Fraunhofer Portugal AICOS, Rua Alfredo Allen 455/461, 4200-135 Porto, Portugal; 3 Faculty of Psychology and Education, Universidade Lusófona—Centro Universitário do Porto, Rua Augusto Rosa, 24, 4000-098 Porto, Portugal; 4 RISE-Health@UPT, Portucalense University, Rua Dr. António Bernardino de Almeida, 541, 4200-072 Porto, Portugal; ana.bartolo@upt.pt; 5 Department of Education and Psychology, University of Aveiro, Campus Universitário de Santiago, 3810-193 Aveiro, Portugal; liliana.m.loureiro@ua.pt; 6 RISE-Health: Health Research Network, Department of Psychology and Education, Faculty of Human and Social Sciences, University of Beira Interior, Estrada do Sineiro, 6200-209 Covilhã, Portugal; ana.carla.torres@ubi.pt; 7 William James Center for Research—WJCR, Department of Education and Psychology, University of Aveiro, Campus Universitário de Santiago, 3810-193 Aveiro, Portugal

**Keywords:** acceptability, cancer-related cognitive impairment, cancer survivors, cognitive rehabilitation, patient-reported outcomes, psychometrics, scale development, Theoretical Framework of Acceptability

## Abstract

**Background/Objectives**: Cancer-related cognitive impairment (CRCI) is a prevalent survivorship concern associated with functional limitations and reduced quality of life. Although cognitive rehabilitation shows beneficial effects, implementation depends not only on efficacy but also on survivors’ anticipated willingness to engage. No psychometrically validated instrument currently exists to assess prospective acceptability of cognitive rehabilitation among cancer survivors. This study aimed to: (1) develop the Acceptability of Cognitive Rehabilitation Interventions Survey–Cancer Survivors (ACRIS-CS); (2) examine its structural validity (factor structure), internal consistency, and construct validity; (3) describe prospective acceptability levels; and (4) identify independent predictors. **Methods**: A cross-sectional study was conducted with 154 cancer survivors reporting cognitive complaints. The ACRIS-CS was developed based on the Theoretical Framework of Acceptability. Structural validity was examined using exploratory factor analysis (EFA); internal consistency was assessed with Cronbach’s alpha; and construct validity was evaluated through hypothesis-driven correlational analyses. Multiple linear regression was used to identify independent predictors of prospective acceptability. **Results:** EFA supported a three-factor structure explaining 68.7% of total variance (KMO = 0.847, Bartlett’s χ^2^ (55) = 864.431, *p* < 0.001). The total scale demonstrated good internal consistency (α = 0.85; subscales α = 0.73–0.85), and construct validity was supported. Mean prospective acceptability indicated overall moderate-to-high levels. Being in active treatment (β = −0.248, *p* = 0.001) and having greater prior knowledge of CRCI (β = 0.240, *p* = 0.002) were independently associated with prospective acceptability (adjusted R^2^ = 0.113). **Conclusions**: The ACRIS-CS demonstrated preliminary evidence of structural validity, internal consistency, and construct validity. Prospective acceptability appears to be influenced by treatment status and prior knowledge of CRCI, supporting the need for further confirmatory validation before routine clinical implementation.

## 1. Introduction

Cancer-related cognitive impairment (CRCI) is a common and clinically relevant survivorship concern that can persist across the cancer continuum [1,2,3]. These cognitive difficulties—often described as mental cloudiness, reduced cognitive abilities, and slower processing speed [4]—may affect memory, attention, executive functioning, and overall information-processing abilities [5,6]. CRCI can emerge before treatment, during therapy, or long after completion [7,8,9], with severity ranging from mild to severe [10]. Although some individuals recover spontaneously [11], others experience persistent impairment lasting up to 20 years post-treatment [12]. Prevalence estimates range from 44% to 75% [7,9,13,14], and the consequences for psychosocial well-being [15,16], daily functioning, interpersonal relationships, employment, and social participation are substantial [1,3,16,17,18].

These impacts underscore the need for accessible and effective interventions to support cognitive functioning in survivorship [19]. Reflecting this priority, the World Health Organization’s Package of Interventions for Rehabilitation for Cancer identifies cognitive functioning as a key rehabilitation component of survivorship care [20]. Among non-pharmacological approaches—including physical exercise, mind–body interventions, and multimodal programs [8,21,22,23,24,25]—cognitive rehabilitation has demonstrated particularly strong evidence for reducing cognitive complaints, improving objective cognitive performance, and enhancing quality of life [14,21,25,26,27,28,29,30,31,32,33]. Cognitive rehabilitation can be delivered individually or in groups [8,33], in person or remotely [26,31,33], and typically combines psychoeducation with skills training and compensatory strategy training [26,31,32]. Despite demonstrated efficacy and high levels of patient satisfaction [27,31], implementation remains challenging due to practical barriers, including session and intervention lengths, time commitment, and high attrition rates (3–42%) [33,34], raising concerns about feasibility and sustainability in routine cancer survivorship care [19].

Although cognitive rehabilitation has demonstrated benefits internationally [27], it remains underutilized in Portugal, where CRCI care pathways are still emerging [35]. Successful implementation in routine survivorship care requires not only evidence of clinical effectiveness but also an understanding of survivors’ anticipated willingness to engage with such interventions [36,37,38]. Acceptability—defined within the Theoretical Framework of Acceptability (TFA) as the extent to which an intervention is perceived as appropriate based on individuals’ cognitive and emotional responses [37]—captures this dimension. The TFA conceptualizes acceptability as comprising seven interrelated constructs: affective attitude, burden, perceived effectiveness, ethicality, intervention coherence, opportunity costs, and self-efficacy. Importantly, acceptability can be assessed prospectively (prior to intervention exposure), concurrently, or retrospectively (following experience). Prospective acceptability is particularly relevant in early stages of service development and implementation, as it may shape initial engagement decisions and inform intervention design [37,39,40].

Despite its relevance, prospective acceptability has rarely been examined in the context of cognitive rehabilitation for CRCI. Existing studies have largely focused on retrospective satisfaction and feasibility following intervention participation [31], leaving a limited understanding of how survivors perceive cognitive rehabilitation prior to engagement [19]. Identifying factors that shape prospective acceptability is essential to tailoring interventions, informing implementation strategies, and developing responsive survivorship care models [41,42,43]. Although the exploration of acceptability has been addressed in prior Portuguese research, existing approaches have relied primarily on study-specific, purpose-built questionnaires rather than psychometrically validated instruments. For example, Sousa et al. [44] examined acceptability and preferences regarding a telephone-based psychological intervention among cancer survivors in the Azores using an ad hoc questionnaire developed for that study, providing valuable descriptive insights but without formal psychometric validation or grounding in a comprehensive theoretical framework. To our knowledge, no instrument available in Portuguese has been systematically developed and psychometrically validated to assess prospective acceptability of cognitive rehabilitation interventions among cancer survivors, particularly in alignment with the seven domains of the TFA. Given that acceptability is likely to vary according to intervention content, delivery format, and the cognitive and behavioral demands placed on participants, a context-specific and theoretically grounded measure is warranted.

Accordingly, this study aimed to: (1) develop a theoretically grounded measure of prospective acceptability of cognitive rehabilitation in cancer survivors (the Acceptability of Cognitive Rehabilitation Interventions Survey–Cancer Survivors; ACRIS-CS); (2) examine its structural validity, internal consistency, and construct validity; (3) describe levels of prospective acceptability in a clinical sample of cancer survivors reporting cognitive complaints; and (4) identify independent predictors of prospective acceptability.

## 2. Materials and Methods

### 2.1. Study Design and Ethical Approval

This cross-sectional study was conducted within the “CanCOG^®^—Cognitive Rehabilitation in Cancer” project in Portugal, which aims to develop a cognitive rehabilitation program for non-central nervous system (non-CNS) cancer survivors. Ethical approval was obtained from the Ethics and Deontology Committee of the University of Aveiro (approval numbers: 30-CED/2019/22 January 2020, and 30-CED/2021/6 October 2021). All participants provided electronic informed consent prior to accessing the questionnaire. Data were collected using a web-based, self-administered LimeSurvey^®^ (Version 5.6) [45] questionnaire hosted on the secure servers of the University of Aveiro, Portugal. The study was conducted in accordance with the Declaration of Helsinki [46] and ethical guidelines of the American Psychological Association [47] and Portuguese Psychologists Association [48]. Grammarly (https://www.grammarly.com) was used to ensure the grammatical accuracy of the document.

### 2.2. Participants and Recruitment

Participants were Portuguese adults aged 18–65 years who: (1) identified as non-CNS cancer survivors (from diagnosis onwards, including those undergoing active treatment [49,50]); (2) were undergoing or had completed cancer treatment; (3) reported cognitive difficulties following diagnosis (i.e., responding “Yes” to the screening question: “Have you been experiencing any cognitive difficulties since being diagnosed with and treated for cancer [e.g., difficulties with memory, attention, concentration, reasoning, decision-making, or planning]?” [19,51,52]); and (4) were able to complete an online questionnaire in Portuguese. Exclusion criteria included: (1) self-reported psychiatric or communication disorders or other severe medical conditions; (2) brain/CNS metastasis; (3) diagnosed brain injury or neurological disease (e.g., stroke, head injury, epilepsy, dementia, Parkinson’s disease, multiple sclerosis), or (4) substance or alcohol abuse, given their potential impact on cognitive functioning [19].

Participants were recruited between September 2022 and April 2023 via: (1) social media advertisements through survivor and patient association pages; (2) email invitations distributed through national cancer associations; (3) notices posted on a research-center webpage; (4) direct email contact with prior study participants who had consented to re-contact; and (5) snowball sampling.

### 2.3. Instrument Development: ACRIS-CS

**Figure 1:**
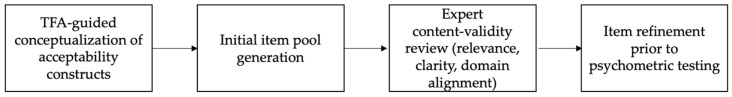
Overview of the development process of the ACRIS-CS guided by the TFA.

The development of the ACRIS-CS followed a structured, theory-informed process guided by the TFA and informed by established best-practice recommendations for scale development and psychometric evaluation [53,54]. This process encompassed conceptualization of acceptability constructs, initial item pool generation, expert content validity review, and item refinement prior to psychometric testing (Figure 1).

#### 2.3.1. Conceptual Framework

The ACRIS-CS is a self-administered instrument developed by the research team to assess prospective acceptability of cognitive rehabilitation in the oncology context. Its development was grounded in the seven theoretical domains of the TFA: (1) affective attitude (emotional responses to the intervention), (2) burden (perceived effort required to participate), (3) perceived effectiveness (expected benefits), (4) ethicality (fit with personal values), (5) intervention coherence (understanding of the intervention), (6) opportunity costs (perceived sacrifices associated with participation), and (7) self-efficacy (confidence in one’s ability to engage in the intervention) [37,39].

#### 2.3.2. Item Generation and Content Validation

An initial pool of items was generated based on the TFA domains and informed by adaptation of items from a previously developed Portuguese oncology acceptability questionnaire [44,55]. Items were reworded to reflect prospective judgments regarding participation in a structured cognitive rehabilitation program. An expert panel comprising four specialists in psycho-oncology and cognitive rehabilitation reviewed the preliminary item pool for theoretical alignment with the TFA domains, content relevance, clarity of wording, and appropriateness for cancer survivorship contexts. Based on expert feedback, items were refined through iterative revision to improve clarity and domain coverage and to remove redundancies.

At the time of data collection, no cognitive rehabilitation program was being offered to participants; therefore, acceptability ratings reflect hypothetical prospective judgments based on a standardized description of a cognitive rehabilitation program. This approach is consistent with the conceptualization of prospective acceptability within the TFA, which explicitly refers to judgments formed prior to intervention exposure. The standardized description outlined the main components, delivery format, and time commitment of a typical cognitive rehabilitation program (see Appendix A). Appendix A presents the final version of the ACRIS-CS in European Portuguese, along with an English translation provided for reference purposes only (not formally validated). The final instrument comprised 11 items rated on a 5-point Likert scale (1 = strongly disagree to 5 = strongly agree). Negatively worded items were reverse-scored so that higher values reflected greater prospective acceptability.

### 2.4. Additional Measures

#### 2.4.1. Sociodemographic and Clinical Variables

Participants reported age, sex, marital status, education, employment status, cancer type, stage, treatments, and prior psychological support.

#### 2.4.2. Anxiety and Depression

Anxiety and depressive symptoms were assessed using the Hospital Anxiety and Depression Scale (HADS) [56,57], which comprises 14 items across two subscales (anxiety and depression). Items are rated on 4-point Likert scales (0–3), with higher scores indicating greater symptom severity. Anxiety and depression levels can be classified as: “normal” (0–7), “mild” (8–10), “moderate” (11–14), and “severe” (15–21). The Portuguese validation study showed good psychometric properties [56]. In the present sample, internal consistency was good for both subscales (α = 0.83 for anxiety; α = 0.81 for depression).

#### 2.4.3. Perceived Cognitive Impairment

Perceived cognitive functioning was assessed using the Perceived Cognitive Impairment (CogPCI) subscale of the Portuguese version of the Functional Assessment of Cancer Therapy-Cognitive Function-Version 3 (FACT-Cog-v3) [13,58,59]. This subscale includes 20 items rated on a 5-point Likert scale (0 = never to 4 = several times a day), with higher scores indicating better perceived cognitive functioning. A cut-off score of <51 represents clinically significant perceived cognitive impairment [13]. The Portuguese validation studies demonstrated strong psychometric properties [13,59]. In the present study, internal consistency was excellent (α = 0.95).

#### 2.4.4. Knowledge of CRCI

Pre-existing knowledge of CRCI was assessed using a single item adapted from prior work [60,61]: “How familiar are you with the idea and/or aware that cognitive difficulties can be a secondary symptom of cancer and/or cancer treatments?”. Responses ranged from 0 (“not at all familiar”) to 4 (“totally familiar”).

### 2.5. Statistical Analysis

All analyses were conducted using IBM SPSS Statistics (Version 30.0.0; IBM Corp., Armonk, NY, USA). Statistical significance was set at *p* < 0.05 (two-tailed). Descriptive statistics were computed to characterize the sample. Distributional properties were examined using skewness and kurtosis indices (acceptable range: −2 to +2) and visual inspection of histograms and Q–Q plots.

As the online survey was structured across multiple pages with mandatory completion of each questionnaire module prior to progression, there were no missing data within completed modules. Only participants with complete ACRIS-CS data were included in the psychometric analyses. Because completion was mandatory within each module but not across all modules, sample sizes varied across measures.

The dimensional structure of the ACRIS-CS was examined using exploratory factor analysis (EFA), with principal component analysis (PCA) as the extraction method and oblique (promax) rotation. PCA was selected as an initial exploratory method appropriate for preliminary scale development and dimensional structure exploration. Sampling adequacy was assessed using the Kaiser–Meyer–Olkin (KMO ≥ 0.8 considered acceptable) measure and Bartlett’s test of sphericity (*p* < 0.05). The recommended sample size for EFA is at least seven participants per item, with a minimum absolute sample size of 100 [62], which supports the adequacy of the data for dimensional structure exploration. Factor retention was guided by eigenvalues greater than 1.0, inspection of the scree plot, and conceptual interpretability, with a minimum of three items per retained factor where possible. Items with standardized loadings ≥ 0.40 were considered salient, and cross-loadings ≥ 0.30 were examined for theoretical coherence. Communalities below 0.30 were scrutinized and retained when conceptually aligned with the theoretical framework. Composite scores were computed by averaging item responses for each factor and for the total scale after reverse-scoring negatively worded items. Mean scores were linearly transformed to a 0–100 scale to facilitate interpretation and comparability across subscales. For interpretive purposes, standardized scores were mapped onto the original Likert response options, with values of 0, 25, 50, 75, and 100 corresponding to “totally disagree”, “disagree”, “neutral”, “agree”, and “totally agree”, respectively.

Internal consistency was assessed using Cronbach’s alpha coefficients for each factor and for the total scale (≥0.70: acceptable; ≥0.80: good; ≥0.90: excellent [63]), alongside corrected item–total correlations (≥0.30 considered satisfactory).

Construct validity was examined using hypothesis-driven correlational analyses. Pearson correlation coefficients were used for associations between continuous variables, and point-biserial correlations for dichotomous variables. Correlation magnitudes were interpreted according to Cohen’s [64] guidelines (small: *r* = 0.10; medium: *r* = 0.30; large: *r* = 0.50).

Multiple linear regression analysis was conducted to identify independent predictors of prospective acceptability (ACRIS-CS total standardized score as the dependent variable). Predictors significantly associated with acceptability in bivariate analyses (*p* < 0.05) were entered into the model, and a backward elimination approach was used to derive a parsimonious model. Categorical predictors with more than two levels were dummy-coded. Regression assumptions were evaluated through inspection of standardized residual plots (linearity and homoscedasticity), histograms and Q–Q plots (normality of residuals), the Durbin–Watson statistic (acceptable range: 1.5–2.5), and tolerance (>0.20) and variance inflation factor (VIF < 5) indices (multicollinearity). Model fit was reported using adjusted R^2^, and results are presented as unstandardized coefficients (B), standardized beta coefficients (β), 95% confidence intervals (CI), and *p*-values.

## 3. Results

### 3.1. Participant Characteristics

**Figure 2:**
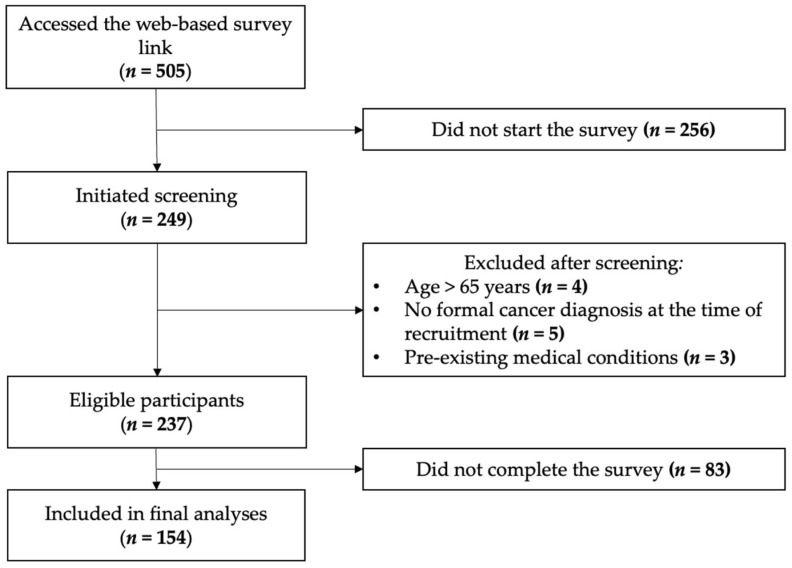
STROBE flow diagram of study participants.

Figure 2 summarizes the participant flow. Of the 505 individuals who accessed the survey link, 249 initiated screening, 237 met eligibility criteria, and 154 completed the survey and were included in the final analyses.

As shown in Table 1, participants had a mean age of 47.3 years (*SD* = 9.0; range = 18–65) and were predominantly female (94.8%). Most were married or in a de facto relationship (70.1%), had completed university education (63.8%), and were employed or self-employed (61.6%).

Breast cancer was the most frequent diagnosis (72.7%), and approximately half of the participants were undergoing active cancer treatment at the time of assessment (49.4%), while 50.6% were in follow-up. Among those currently undergoing treatments, hormone therapy was the most common modality (68.4%), followed by immunotherapy (17.1%) and chemotherapy (11.8%). Regarding disease characteristics, 48.1% of participants had localized cancer (stages 0–II) and 38.3% had non-localized disease (stages III–IV). The mean time since diagnosis was 5.7 years (*SD* = 6.2; range = 1–48).

With respect to psychosocial functioning, 59.1% reported previous or current psychological counselling, and 45.5% reported current use of psychotropic medication. Participants reported mean anxiety levels within the mild range according to established HADS cut-off scores (HADS-A: *M* = 10.0, *SD* = 4.2) and mean depressive symptom levels within the non-clinical range (HADS-D: *M* = 6.9, *SD* = 4.0). Perceived cognitive impairment was prevalent, with mean FACT-Cog-v3 CogPCI scores falling well below the established clinical cut-off for significant cognitive complaints (*M* = 37.8, *SD* = 16.8). Regarding prior knowledge of CRCI, 40.2% of participants reported being not at all or only slightly familiar with the concept, whereas 48.7% reported being familiar or totally familiar.

### 3.2. Structural Validity of the ACRIS-CS

EFA using PCA with oblique (promax) rotation indicated good sampling adequacy (KMO = 0.847) and a significant Bartlett’s test (ꭕ^2^ (55) = 864.431, *p* < 0.001), supporting the suitability of the correlation matrix for factor analysis. A three-factor solution was retained based on eigenvalues > 1.0, inspection of the scree plot, and conceptual interpretability (Table 2). The three-factor solution accounted for 68.7% of the total variance (Factor 1: 44.2%; Factor 2: 15.1%; Factor 3: 9.5%). All items demonstrated salient factor loadings (≥0.40). Item 10 showed cross-loading across two factors and was retained based on conceptual coherence with the factor content. Although Item 9 presented a comparatively lower communality, it was retained due to its conceptual relevance within the TFA domain of ethicality, which is theoretically central to the construct of acceptability and would have been underrepresented if excluded from the scale. Standardized factor loadings and communalities are reported in Table 2.

For interpretative purposes, the extracted components are hereafter referred to as factors. Based on the pattern of loadings and the conceptual interpretation, the three factors were labeled as follows: Factor 1—Affective Attitude and Outcome Expectancies, reflecting the integration of emotional responses toward cognitive rehabilitation (e.g., feeling that participation would be positive) and expectations of its effectiveness; Factor 2—Understanding and Capability, representing participants’ comprehension of how the intervention works, perceived benefits, compatibility with personal values, and confidence in their ability to complete the required tasks; Factor 3—Perceived Burden, capturing the perceived effort, time, and disruption required to participate in a cognitive rehabilitation program.

### 3.3. Reliability (Internal Consistency)

The ACRIS-CS demonstrated acceptable internal consistency for the total scale (Cronbach’s α = 0.84). Subscale internal consistency was acceptable to good: Factor 1 (α = 0.85), Factor 2 (α = 0.73), and Factor 3 (α = 0.75) (Table 3). Corrected item–total correlations ranged from 0.255 to 0.704, and deletion of any item did not result in meaningful improvements in alpha values, supporting adequate internal coherence of the scale.

### 3.4. Construct Validity

Construct validity was examined using hypothesis-driven correlational analyses to evaluate theoretically expected patterns of association between prospective acceptability and relevant sociodemographic, clinical, psychological, and cognitive variables. In line with theoretical expectations, prospective acceptability (ACRIS-CS total standardized score) was significantly associated with treatment status (*r* = −0.259, *p* = 0.001) and prior knowledge of CRCI (*r* = 0.251, *p* = 0.002), both with small-to-medium correlations. Specifically, participants who had completed active treatment and those with greater prior knowledge of CRCI reported higher levels of prospective acceptability.

In contrast, prospective acceptability was not significantly associated with age, sex, education level, time since diagnosis, history of psychological counselling, current psychotropic medication use, anxiety, depressive symptoms, or perceived cognitive impairment (all *p* > 0.05). This pattern of findings supports the discriminant validity of the ACRIS-CS with respect to these variables and is consistent with the conceptualization of acceptability as a construct primarily reflecting intervention-related cognitions and attitudes rather than general demographic or psychological characteristics.

### 3.5. Prospective Acceptability Levels

On the standardized 0–100 scale, the mean prospective acceptability total score was *M* = 67.4, *SD* = 12.1, with a median of 67.05 (IQR = 59.1–75.0). According to the predefined mapping of standardized scores to the original Likert response options, these values correspond to moderate-to-high prospective acceptability (i.e., mean item responses approximating “agree”). Mean standardized subscale scores were 75.4 (SD = 13.4) for Factor 1 (Affective Attitude and Outcome Expectancies), 63.2 (SD = 16.1) for Factor 2 (Understanding and Capability), and 49.4 (SD = 20.8) for Factor 3 (Perceived Burden). A total of 21.4% of participants scored ≥ 75.0, corresponding to mean responses of “agree” or higher, indicating high prospective acceptability among a substantial proportion of the sample. No marked floor or ceiling effects were observed.

Item-level response distributions are shown in Table 3. The highest levels of agreement (“agree” and “strongly agree” combined) were observed for items reflecting expected usefulness and positive attitudes toward cognitive rehabilitation (e.g., item 3: 88.3%; item 1: 86.3%; item 2: 82.5%). The highest levels of disagreement were observed for items reflecting perceived burden and disruption to daily routines (e.g., item 11: 35.1%; item 5: 30.5%).

### 3.6. Predictors of Prospective Acceptability

Multiple linear regression analysis was conducted to identify independent predictors of prospective acceptability (ACRIS-CS total standardized score). The final model explained 11.3% of the variance in prospective acceptability (adjusted R^2^ = 0.113, *F*(2, 151) = 10.736, *p* < 0.001). In the final model, treatment status (β = −0.248, *p* = 0.001) and prior knowledge of CRCI (β = 0.240, *p* = 0.002) emerged as independent predictors of prospective acceptability. Specifically, survivors in active treatment reported higher acceptability compared to those who had completed active treatment (B = −2.636, 95% CI [−4.234, −1.039]), and greater prior knowledge of CRCI was associated with higher acceptability (B = 2.598, 95% CI [0.970, 4.227]). Full regression results are presented in Table 4.

## 4. Discussion

This study describes the development and preliminary psychometric evaluation of the ACRIS-CS, a theoretically grounded instrument designed to assess the prospective acceptability of cognitive rehabilitation among cancer survivors.

The EFA (operationalized using PCA) supported a three-factor structure, providing preliminary evidence of structural validity. Although the ACRIS-CS was originally grounded in the seven domains of the TFA, the three broader factors identified empirically reflect higher-order patterns in how survivors evaluated cognitive rehabilitation. Similar simplifications of theoretically multidimensional acceptability constructs have been reported in healthcare intervention research [65], suggesting that, in applied contexts, multiple TFA domains may operate in an integrated manner. The factor labelled Affective Attitude and Outcome Expectancies captured both emotional valence and perceived effectiveness, indicating that survivors’ positive feelings toward rehabilitation are closely intertwined with beliefs about its potential benefits. The Understanding and Capability factor integrated intervention coherence, ethical fit, perceived benefits, and self-efficacy, reflecting a broader cognitive readiness to engage. In contrast, Perceived Burden emerged as a distinct dimension, capturing anticipated effort, time demands, and disruption to daily routines. The separation of burden from positive appraisals underscores that survivors may simultaneously view cognitive rehabilitation as valuable while anticipating practical barriers to participation, a pattern consistent with implementation science frameworks emphasizing burden as an independent determinant of engagement [36].

The ACRIS-CS demonstrated good internal consistency and theoretically coherent associations with prior knowledge of CRCI and treatment status, providing preliminary support for construct validity. Overall levels of prospective acceptability were moderate to high, with a substantial proportion of participants indicating strong receptivity to cognitive rehabilitation. However, the discrepancy between high affective/expectancy scores and moderate perceived burden highlights a critical implementation challenge. Although survivors recognize the value of cognitive rehabilitation, anticipated logistical demands may hinder engagement and sustained participation. This pattern is consistent with previously reported attrition rates in cognitive rehabilitation trials [34] and underscores the importance of intervention design features that reduce perceived burden, such as flexible scheduling, remote delivery, and modular or asynchronous formats [26,31]. Emerging evidence supports the feasibility and effectiveness of telehealth-based cognitive rehabilitation [66,67,68], suggesting that technology-enabled delivery may help reconcile perceived benefits with practical feasibility.

Regression analyses indicated that prospective acceptability was higher among survivors undergoing active treatment and among those with greater prior knowledge of CRCI, jointly explaining a modest proportion of variance. These findings support the notion that acceptability is multifactorial and shaped by both illness phase and informational context. Greater acceptability during active treatment may reflect heightened symptom salience and increased engagement with healthcare services, facilitating openness to supportive interventions [8,69]. The association with CRCI knowledge highlights the importance of patient education in shaping receptivity to cognitive rehabilitation. Integrating CRCI information into routine oncology care and survivorship planning may therefore represent a modifiable lever to enhance acceptability and early uptake [29,30]. Nevertheless, the modest variance explained highlights that acceptability is likely influenced by additional, unmeasured psychosocial and contextual determinants (e.g., social support, financial constraints, accessibility, prior rehabilitation experiences), which should be examined in future research alongside more comprehensive psychometric and longitudinal validation of the ACRIS-CS.

### 4.1. Limitations

Several limitations should be acknowledged. This cross-sectional design precludes causal inference and assessment of temporal stability. The sample was predominantly female and largely comprised breast cancer survivors, limiting generalizability to other cancer populations. Given that gender and cancer type may influence illness representations and attitudes toward rehabilitation, the predominantly female breast cancer sample may have shaped the observed acceptability patterns. Although structural validity, internal consistency, and construct validity were examined, additional psychometric properties, including test–retest reliability, convergent validity, and predictive validity for actual intervention uptake, remain to be established. Content validity was informed by expert review but not formally assessed with cancer survivors, and future qualitative work should incorporate patient perspectives to ensure comprehensive coverage of survivor-relevant concerns. The reliance on self-report measures may also introduce response bias. Finally, this study focused on prospective acceptability; future research should examine both prospective and experienced acceptability to capture the full trajectory of engagement with cognitive rehabilitation.

### 4.2. Implications and Future Research

The ACRIS-CS is intended for prospective use, that is, prior to survivors initiating cognitive rehabilitation, to capture their initial receptivity, perceived benefits, and anticipated barriers to engagement. The prospective assessment of acceptability is particularly relevant in oncology rehabilitation contexts, as acceptability has been identified as a key determinant of intervention uptake, adherence, and implementation success in healthcare settings [37,70]. By identifying survivors who anticipate high burden or express lower initial receptivity before intervention delivery, the ACRIS-CS may support clinicians and rehabilitation teams in tailoring preparatory strategies, such as targeted psychoeducation, expectation management, and shared decision-making, to enhance engagement [37,71].

In clinical practice, the ACRIS-CS may be administered by psychologists, oncology healthcare providers, or rehabilitation professionals as part of routine survivorship care, pre-intervention screening, or implementation protocols. The standardized 0–100 scoring system enhances usability by providing intuitive and easily interpretable values, allowing clinicians to rapidly identify survivors with lower overall acceptability or elevated concerns within specific domains. This facilitates early triage and the design of individualized support strategies prior to intervention initiation, such as flexible scheduling, remote delivery options, or additional clarification regarding intervention demands and expected benefits.

Beyond the total score, interpretation of the ACRIS-CS at the factor-profile level may offer clinically meaningful insights into the specific drivers of acceptability for each individual. High scores on the Affective Attitude/Outcome Expectancies and Understanding/Capability factors may indicate stronger readiness to engage, whereas elevated Perceived Burden scores may signal practical barriers requiring targeted implementation strategies. This profile-based interpretation supports more personalized intervention planning and aligns with stepped-care and implementation science frameworks that emphasize the tailoring of interventions to individual readiness and contextual constraints.

In research contexts, the ACRIS-CS provides a standardized measure to examine predictors of prospective acceptability and to support implementation science studies investigating readiness for cognitive rehabilitation in oncology settings. The instrument may be used to evaluate acceptability as an early implementation outcome, to compare delivery formats (e.g., in-person vs. remote interventions), and to examine how modifications to intervention design influence anticipated burden and perceived benefit. Given the documented challenges in engagement and adherence to cognitive rehabilitation interventions among cancer survivors [27,72], particularly when perceived burden is high or intervention relevance is unclear, systematic assessment of acceptability may inform the development of more responsive and sustainable service models.

Although PCA was used as an initial extraction method for exploratory purposes in this preliminary validation study, future research should further examine the latent structure of the ACRIS-CS using common factor analytic models (e.g., principal axis factoring) and confirmatory factor analysis in independent samples. Additional psychometric evaluation is warranted to establish test–retest reliability and to examine convergent and predictive validity, particularly in relation to actual uptake, adherence, and clinical outcomes of cognitive rehabilitation. Longitudinal designs will be essential to investigate the temporal stability of prospective acceptability and its evolution over time in relation to symptom trajectories and intervention exposure. Moreover, validation in more diverse and representative samples, including greater representation of male survivors and non-breast cancer populations, is needed to strengthen generalizability. Finally, qualitative research incorporating survivor perspectives may further elucidate contextual and experiential determinants of acceptability, informing ongoing refinement of the instrument and the development of targeted implementation strategies.

## 5. Conclusions

The ACRIS-CS demonstrated promising preliminary evidence of structural validity, internal consistency, and construct validity as a measure of prospective acceptability of cognitive rehabilitation among cancer survivors. Further confirmatory validation and longitudinal research are warranted prior to widespread clinical implementation. By enabling systematic assessment of perceived benefits, burden, and readiness to engage, the ACRIS-CS may support more tailored, patient-centered approaches to implementing cognitive rehabilitation within survivorship care pathways.

## Figures and Tables

**Table 1:** Sample characteristics (*N* = 154).

Characteristics	*N*	*n*	%
**Age at enrolment in years (*M* ± *SD*; range)**	154	47.3 ± 9.0; 18–65	
**Sex**	154		
Male		8	5.2
Female		146	94.8
**Marital status**	154		
Married/de facto relationship		108	70.1
Single		24	15.6
Divorced/separated		18	11.7
Widowed		4	2.6
**Education level**	152		
Primary school (completed or unfinished)		7	4.6
Middle school		12	7.9
High school		36	23.7
University		97	63.8
**Employment status**	151		
Employed/Self-employed (full- or part-time)		93	61.6
Unemployed		17	11.3
Sick leave		29	19.2
Retired		10	6.6
Student		2	1.3
**Type of cancer**	154		
Breast		112	72.7
Ovarian		8	5.2
Colorectal		7	4.5
Leukemia		2	1.3
Hodgkin Lymphoma		7	4.5
Non-Hodgkin Lymphoma		6	3.9
Other cancer types ^1^		12	7.6
**Cancer staging**	154		
Localized cancer (stages 0, I, II)		74	48.1
Non-localized cancer (stages III, IV)		59	38.3
Unknown information		21	13.6
**Time since diagnosis in years (*M* ± *SD*; range)**	154	5.7 ± 6.2; 1–48	
**Cancer treatment history**	154		
Surgery		123	79.9
Chemotherapy		137	89.0
Radiotherapy		106	68.8
Hormone therapy		82	53.2
Immunotherapy		26	16.9
Targeted therapy		10	6.5
Stem cell transplant		3	1.9
**Current treatment status**	154		
Undergoing active treatment		76	49.4
Follow-up (completed active treatment)		78	50.6
**Current cancer treatment**	76		
Chemotherapy		9	11.8
Radiotherapy		4	5.3
Hormone therapy		52	68.4
Immunotherapy		13	17.1
Targeted therapy		3	3.9
**Psychological counseling (previous or current) (Yes)**	154	91	59.1
**Currently taking psychotropic medication ^2^ (Yes)**	154	70	45.5
**Psychological distress (HADS)**	117		
Anxiety (*M* ± *SD*; range)		10.0 ± 4.2; 2–21	
Depression (*M* ± *SD*; range)		6.9 ± 4.0; 0–19	
**Perceived cognitive impairment (CogPCI FACT-Cog-v3) (*M* ± *SD*; range)**	118	37.8 ± 16.8; 3–76	
**Knowledge of CRCI**	154		
No, not at all [familiar] + Slightly [familiar]		62	40.2
Neutral		17	11.0
Yes, [familiar] + Totally [familiar]		75	48.7

^1^ Note: Includes multiple myeloma, lung, thyroid, bone, stomach, skin, cervical, endometrial, head and neck cancer, and leiomyosarcoma. ^2^ Includes sedatives and hypnotics, antidepressants, anxiolytics, and antipsychotics.

**Table 2:** Exploratory factor analysis of the ACRIS-CS (principal component analysis with promax rotation): standardized factor loadings, communalities, item–total correlations, and internal consistency indices.

Item	Corrected Item-Total Correlation	Cronbach’s Alpha if an Item is Deleted	F1 ^1^	F2 ^2^	F3 ^3^	h^2 4^
3	0.673	0.817	**0.962**	−0.092	0.032	0.852
2	0.662	0.817	**0.929**	−0.071	0.039	0.815
1	0.704	0.815	**0.830**	0.051	0.050	0.765
7	0.624	0.821	**0.644**	0.284	−0.109	0.644
9	0.255	0.855	**0.621**	−0.189	−0.089	0.283
10	0.685	0.815	**0.471**	0.461	−0.032	0.653
8	0.429	0.834	−0.188	**0.968**	−0.175	0.685
4	0.531	0.826	0.021	**0.712**	0.047	0.554
6	0.670	0.813	−0.013	**0.691**	0.309	0.734
11	0.398	0.838	0.037	−0.104	**0.910**	0.777
5	0.381	0.843	−0.095	0.041	**0.898**	0.795
% Explained Variance			44.2	15.1	9.5	-
Cronbach’s Alpha (α)			0.85	0.73	0.75	-
Mean (*SD*), range (standardized 0–100 scores)			75.4 (13.4), 42–100	63.2 (16.1), 17–100	49.4 (20.8), 0–100	-
ACRIS-CS Total Score, Mean (*SD*), range (standardized 0–100 scores)			67.4 (12.1), 34–100			

^1^ Factor 1: Affective Attitude and Outcome Expectancies; ^2^ Factor 2: Understanding and Capability; ^3^ Factor 3: Perceived burden; ^4^ h^2^ = communality. Factor loadings ≥ 0.40 are considered salient and are highlighted in bold. Corrected item–total correlations and Cronbach’s alpha if deleted items are reported to inform internal consistency.

**Table 3:** Descriptive analysis of the ACRIS-CS scale (*N* = 154).

Item	Item Description	Totally Disagree ^1^	Disagree ^1^	Neutral ^1^	Agree ^1^	Totally Agree ^1^
1	Perceived overall benefit of participating	0 (0)	0 (0)	21 (13.6)	90 (58.4)	43 (27.9)
2	Belief that the program improves cognitive functions	0 (0)	1 (0.6)	26 (16.9)	87 (56.5)	40 (26)
3	Perceived effectiveness in addressing cognitive difficulties	0 (0)	1 (0.6)	17 (11)	95 (61.7)	41 (26.6)
4	Ease of integrating home training exercises into routine	2 (1.3)	14 (9.1)	43 (27.9)	82 (53.2)	13 (8.4)
5	Perceived disruption of daily routine	10 (6.5)	37 (24)	50 (32.5)	50 (32.5)	7 (4.5)
6	Ease of integrating program participation into routine	2 (1.3)	21 (13.6)	58 (37.7)	61 (39.6)	12 (7.8)
7	Expected effectiveness in improving cognitive difficulties	0 (0)	0 (0)	37 (24)	93 (60.4)	24 (15.6)
8	Confidence in ability to complete program tasks	1 (0.6)	5 (3.2)	59 (38.3)	77 (50)	12 (7.8)
9	Alignment of the program with personal beliefs/values	5 (3.2)	9 (5.8)	23 (14.9)	73 (47.4)	44 (28.6)
10	Overall attractiveness of the intervention	0 (0)	2 (1.3)	37 (24)	89 (57.8)	26 (16.9)
11	Perceived time and energy burden	2 (1.3)	52 (33.8)	64 (41.6)	31 (20.1)	5 (3.2)

^1^ *n* (%).

**Table 4:** Multiple linear regression model predicting prospective acceptability of cognitive rehabilitation (ACRIS-CS total standardized score).

Independent Variables	B	β	95%CI	*t*	*p*
Treatment status (completed active treatment = 1)	−2.636	−0.248	−4.234; −1.039	−3.26	0.001
Knowledge of CRCI (familiar/totally familiar = 1)	2.598	0.240	0.970; 4.227	3.15	0.002
Model fit	*F*(2, 151) = 10.736, *p* < 0.001				
adjusted R^2^	0.113				

Note. B = unstandardized coefficient; β = standardized coefficient. Treatment status coded as 1 = completed active treatment, 0 = undergoing active treatment; Knowledge of CRCI coded as 1 = familiar/totally familiar, 0 = not at all/slightly familiar.

## Data Availability

The data that support the findings of this study are available from the corresponding authors upon reasonable request.

## Abbreviations

The following abbreviations are used in this manuscript:

ACRIS-CS	Acceptability of Cognitive Rehabilitation Interventions Survey–Cancer Survivors
CNS	Central nervous system
CogPCI FACT-Cog-v3	Perceived Cognitive Impairment subscale of the Functional Assessment of Cancer Therapy-Cognitive Function-Version 3
CRCI	Cancer-related cognitive impairment
EFA	Exploratory factor analysis
HADS	Hospital Anxiety and Depression Scale
IQR	Interquartile ranges
KMO	Kaiser-Meyer-Olkin
PCA	Principal component analysis
*SD*	Standard deviation
TFA	Theoretical Framework of Acceptability

## Appendix A

### Appendix A.1. European Portuguese Version of the ACRIS-CS

Questionário de Aceitabilidade face às Intervenções de Reabilitação Cognitiva–Sobreviventes de Cancro (ACRIS-CS).

Este questionário avalia a aceitabilidade de intervenções de reabilitação cognitiva entre sobreviventes de cancro. Antes de preencher este questionário, por favor, leia a seguinte descrição dos programas de reabilitação cognitiva:

“A reabilitação cognitiva é uma abordagem em que um profissional de saúde qualificado ajuda os pacientes a desenvolver estratégias compensatórias para gerir dificuldades cognitivas específicas (tais como usar agendas, planificadores ou sistemas de lembretes e aprender técnicas para minimizar distrações). O profissional também fornece informação útil sobre o funcionamento do cérebro, os défices cognitivos e suas consequências na vida diária (psicoeducação). Adicionalmente, a reabilitação cognitiva pode oferecer treino cognitivo (“treino mental”), que envolve exercícios repetidos concebidos para melhorar capacidades cognitivas, tais como a atenção, a memória, a velocidade de processamento e as funções executivas. Estes exercícios são praticados tanto durante as sessões com o profissional como em casa entre sessões.

Os programas de reabilitação cognitiva podem ser realizados individualmente ou em grupo, presencialmente ou remotamente (por meio de videoconferência ou de plataformas online). Os programas abrangem, tipicamente, múltiplas sessões ao longo de várias semanas.”

Com base nesta descrição, por favor, indique o seu nível de concordância (se Discorda Totalmente, Discorda, está Neutro(a), Concorda ou Concorda Totalmente) com cada uma das seguintes afirmações sobre programas de reabilitação cognitiva que podem ou não refletir as suas expectativas ou opiniões.

“Perante dificuldades cognitivas relacionadas com o cancro/tratamentos…”

**Table A1:** ACRIS-CS statements (in European Portuguese).

		Discordo Fortemente	Discordo	Neutro(a)	Concordo	Concordo Fortemente
1	Sinto que a participação num programa de reabilitação cognitiva seria positiva para mim					
2	Reconheço que a participação em programas de reabilitação cognitiva iria melhorar as minhas funções cognitivas					
3	A participação num programa de reabilitação cognitiva seria útil para lidar com as minhas dificuldades cognitivas					
4	Teria facilidade em integrar na minha rotina diária a realização de exercícios de treino cognitivo entre as sessões de um programa de reabilitação cognitiva					
5	Sinto que a participação num programa de reabilitação cognitiva iria comprometer a minha rotina diária					
6	Teria facilidade em integrar na minha rotina diária a participação num programa de reabilitação cognitiva					
7	Os programas de reabilitação cognitiva seriam eficazes para resolver/melhorar as minhas dificuldades cognitivas					
8	Tenho confiança de que seria capaz de realizar todas as tarefas de um programa de reabilitação cognitiva					
9	A participação num programa de reabilitação cognitiva não iria colidir com as minhas crenças e valores					
10	Os programas de reabilitação cognitiva seriam uma opção de intervenção atrativa para mim					
11	Participar num programa de reabilitação cognitiva exigiria de mim muito tempo e energia					

Cotação:

1. Inverter a cotação dos itens 5 e 11 (1 ⟶ 5, 2 ⟶ 4, 3 ⟶ 3, 4 ⟶ 2, 5 ⟶ 1)
2. Calcular as pontuações das subescalas:
  - Fator 1 (Atitude e Benefícios Esperados): Pontuação média dos itens 1, 2, 3, 7, 9 e 10
  - Fator 2 (Compreensão e Capacidade de Participação): Pontuação média dos itens 4, 6 e 8
  - Fator 3 (Sobrecarga Percebida): Pontuação média dos itens 5R e 11R
3. Calcular a pontuação total: Pontuação média de todos os 11 itens (após inversão da cotação)
4. Padronizar para escala 0–100: ((pontuação − 1)/4) × 100

Interpretação: Pontuações mais elevadas indicam maior aceitabilidade.

### Appendix A.2. English Translated Version of the ACRIS-CS [Free Translation, Not Validated]

The following presents the English translation of the ACRIS-CS. The validated European Portuguese version should be used with the European Portuguese-speaking population. Translation to other languages should follow established translation and cultural adaptation procedures (forward translation, back translation, expert review, cognitive debriefing) with subsequent psychometric validation.

Acceptability of Cognitive Rehabilitation Interventions Survey–Cancer Survivors (ACRIS-CS).

This questionnaire assesses the acceptability of cognitive rehabilitation interventions among cancer survivors. Before completing this questionnaire, please read the following description of cognitive rehabilitation programs:

“Cognitive rehabilitation is an approach where a qualified healthcare professional helps patients develop compensatory strategies to manage specific cognitive difficulties (such as using agendas, planners, or reminder systems, and learning techniques to minimize distractions). The professional also provides useful information about brain functioning, cognitive deficits, and their consequences in daily life (psychoeducation). Additionally, cognitive rehabilitation can offer cognitive training (“mental training”), which involves repeated exercises designed to improve cognitive abilities such as attention, memory, processing speed, and executive functions. These exercises are practiced both during sessions with the professional and at home between sessions.

Cognitive rehabilitation programs can be delivered individually or in groups, in person or remotely (via videoconferencing or online platforms). Programs typically span multiple sessions across several weeks.”

Based on this description, please indicate your level of agreement (whether you Totally Disagree, Disagree, are Neutral, Agree, or Totally Agree) with each of the following statements about cognitive rehabilitation programs that may or may not reflect your expectations or opinions.

“Faced with cognitive difficulties related to cancer/treatments…”

**Table A2:** ACRIS-CS statements (in English).

		Totally Disagree	Disagree	Neutral	Agree	Totally Agree
1	I feel that participation in a cognitive rehabilitation program would be positive for me					
2	I recognize that participation in cognitive rehabilitation programs would improve my cognitive functions					
3	Participation in a cognitive rehabilitation program would help address my cognitive difficulties					
4	I would easily integrate cognitive training exercises into my daily routine between sessions of a cognitive rehabilitation program					
5	I feel that participating in a cognitive rehabilitation program would compromise my daily routine					
6	I would easily integrate participation in a cognitive rehabilitation program into my daily routine					
7	Cognitive rehabilitation programs would be effective in resolving/improving my cognitive difficulties					
8	I am confident that I would be able to perform all tasks in a cognitive rehabilitation program					
9	Participation in a cognitive rehabilitation program would not conflict with my beliefs and values					
10	Cognitive rehabilitation programs would be an attractive intervention option for me					
11	Participating in a cognitive rehabilitation program would require a lot of time and energy from me					

Scoring:

1. Reverse-score items 5 and 11 (1 ⟶ 5, 2 ⟶ 4, 3 ⟶ 3, 4 ⟶ 2, 5 ⟶ 1)
2. Calculate subscale scores:
  - Factor 1 (Affective Attitude and Outcome Expectancies): Average score of items 1, 2, 3, 7, 9, and 10
  - Factor 2 (Understanding and Capability): Average score of items 4, 6, and 8
  - Factor 3 (Perceived Burden): Average score of items 5R and 11R
3. Calculate total score: Average score of all 11 items (after reverse-scoring)
4. Standardize to 0–100: ((score − 1)/4) × 100

Interpretation: Higher scores indicate greater acceptability.
